# Optimizing estradiol level for gonadotrophin-releasing hormone antagonist initiation among patients with simple tubal factor infertility

**DOI:** 10.3389/fendo.2022.915923

**Published:** 2022-09-09

**Authors:** Yizhuo Wang, Xiuhua Xu, Ai-min Yang, Jie Zhang, Zhuo-ye Luo, Yan Han, Na Cui, Qian Li, Baojun Shi, Zhi-ming Zhao, Gui-min Hao

**Affiliations:** ^1^ Hebei Key Laboratory of Infertility and Genetics, Hebei Clinical Research Center for Birth Defects, Department of Reproductive Medicine, Second Hospital of Hebei Medical University, Shijiazhuang, China; ^2^ Cardiovascular Platform, Institute of Health and Disease, Hebei Medical University, Shijiazhuang, China

**Keywords:** GnRH antagonist, estradiol, fresh embryo transfer, clinical pregnancy, controlled ovarian hyperstimulation

## Abstract

**Objective:**

The aim of this study is to investigate the optimal estradiol (E_2_) level on the day of gonadotropin-releasing hormone antagonist (GnRH-ant) initiation to maximize the clinical pregnancy rate (CPR) after fresh embryo transfer among patients with simple tubal factor infertility.

**Methods:**

A retrospective cohort study was conducted in the Reproductive Medicine Center, the Second Hospital of Hebei Medical University. A total of 1,493 IVF-ET cycles of patients diagnosed with single tubal factor infertility from August 2016 to August 2021 were included and equally allocated into five distinct groups according to the quintile serum E_2_ levels on the day of GnRH-ant initiation. The five groups had similar baseline data except for antral follicle count.

**Result(s):**

The serum E _2_ level on GnRH-ant initiation day was determined as an independent predictor of clinical pregnancy after adjusting for confounding factors such as age, infertility duration, body mass index, cycle number, antral follicle count, and the number of transferred embryos. Through smooth curve fitting, we found that, with the increase of serum E_2_ levels on the day of GnRH-ant initiation, CPR showed a trend of slight increase and then slight decrease. The maximal CPR was achieved when the serum E_2_ level on GnRH-ant initiation day was 498 pg/ml. When E_2_ was less than 498 pg/ml, the odds ratio (OR) of clinical pregnancy was 1.05 (95% CI: 1.00, 1.11, *P =* 0.0583). When E_2_ was greater than 498 pg/ml, the OR of clinical pregnancy was 0.97 (95% CI: 0.95, 0.98, *P =* 0.0003). Furthermore, CPR remained high when E_2_ was 436.8–658.6 pg/ml but declined significantly by more than 40% when E_2_ was ≥ 894.4 pg/ml (*P <* 0.05).

**Conclusion(s):**

The serum E_2_ level should be considered as an adjuvant parameter for GnRH-ant initiation. The best E_2_ value was 498 pg/ml, and GnRH-ant administration could be recommended to initiate when serum E_2_ was 436.8–658.6 pg/ml. If GnRH-ant was initiated when serum E_2_ was above 894.4 pg/ml, then the CPR after fresh embryo transfer may decline dramatically, and thus, cancellation of fresh embryo transfer and earlier initiation of GnRH-ant in future cycles should be considered.

## Introduction

Preventing luteinizing hormone (LH) surges is key to inducing multiple follicular maturation patterns during controlled ovarian hyperstimulation (COH). As an analog of gonadotropin (Gn)–releasing hormone (GnRH) without biological activity, GnRH antagonist (GnRH-ant) effectively inhibits LH secretion by competitively binding to pituitary GnRH receptors and rapidly blocking the effect of endogenous GnRH (1). Since its first introduction in the 1990s, the GnRH-ant protocol has won increasing popularity and has been recommended over the GnRH agonist protocols by the European Society of Human Reproduction and Embryology (ESHRE) as the first-line COH treatment in the general *in vitro* fertilization (IVF) or intracytoplasmic sperm injection (ICSI) population (2). This is due to several advantages of the GnRH-ant protocol, such as the avoidance of hypo-estrogenic side effects of pituitary downregulation, reduced exogenous Gn dosage and duration, and lower risk of ovarian hyperstimulation syndrome (OHSS) (1).

In general, the patterns of GnRH-ant initiation are classified as “fixed” and “flexible”. In the fixed protocol, the administration of GnRH-ant starts on a predetermined day (usually day 6) of the COH cycle, whereas the initiation of GnRH-ant in the flexible protocol is determined by individual response to each cycle (3). Previous studies comparing both protocols have shown a comparable number of retrieved oocytes, rate of premature LH surge, and pregnancy outcomes, but the flexible protocol was found to be associated with a reduced dosage of GnRH-ant and Gn (3–6).

Despite widespread use, the criteria for GnRH-ant initiation, especially in the flexible protocol, have yet to be standardized, and considerable variations remain among different clinical settings. Conventionally, the most frequently used parameter in flexible protocol was follicular size, whereas hormonal parameters were not recommended (7). However, given that evidence supporting the association between the supraphysiologic estrogen level and adverse pregnancy outcomes keeps piling up (8–10), more attention to the serum estradiol (E_2_) level during COH using the GnRH-ant protocol is warranted. We hypothesized that the serum E_2_ level on the day of GnRH-ant initiation would serve as an independent predictor of clinical pregnancy following COH using the GnRH-ant protocol and subsequent fresh embryo transfer. The objective of this study was to investigate the optimal E_2_ level on the day of GnRH-ant initiation to maximize the clinical pregnancy rate (CPR) after fresh embryo transfer among patients with simple tubal factor infertility.

## Materials and methods

### Study design

This is a retrospective cohort study conducted at the Reproductive Medicine Center, the Second Hospital of Hebei Medical University, from August 2016 to August 2021. This study followed the Strengthening the Reporting of Observational Studies in Epidemiology guidelines and was approved by the Institutional Review Board of the Second Hospital of Hebei Medical University. All methods were performed in accordance with the relevant guidelines and regulations.

### Study subjects

**Figure 1:**
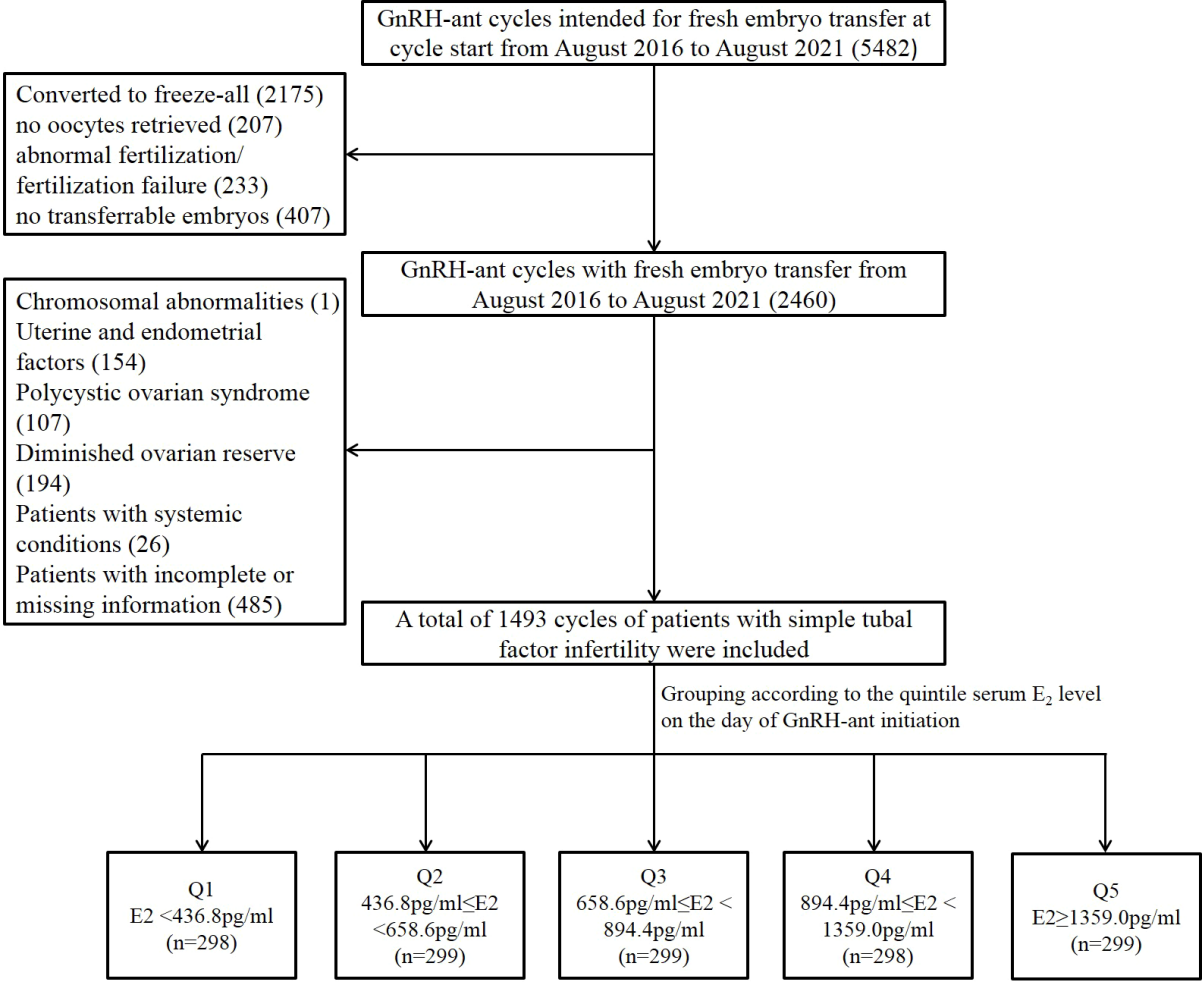
Flow chart of the study population selection and grouping. GnRH-ant, gonadotropin releasing hormone antagonist; E2, estradiol; Q1, quintile 1; Q2, quintile 2; Q3, quintile 3; Q4, quintile 4; Q5, quintile 5.

Cycles were included if they met the following criteria: (i) the GnRH-ant protocol was used; and (ii) intended for fresh embryo transfer at cycle start. The exclusion criteria were as follow: (i) cycles converted to freeze-all (ii) fresh embryo transfer canceled due to all reasons, including no oocytes retrieved, abnormal fertilization, fertilization failure, and no transferrable embryos; (iii) chromosomal abnormalities of either of the couple; (iv) patients with uterine or endometrial conditions affecting pregnancy outcomes, such as uterine malformations, uterine fibroids, adenomyoma, endometriosis, endometrial polyps, intrauterine adhesions, history of endometrial tuberculosis, and inflow of the hydrosalpinx fluid to the uterine cavity; (v) patients with polycystic ovarian syndrome or diminished ovarian reserve [as previously defined (11)]; and (vi) patients with systemic diseases such as diabetes mellitus or thyroid diseases. A total of 485 cycles were excluded because of missing data. Finally, a total of 1,493 cycles of patients with simple tubal factor infertility were included (**Figure 1**). Simple tubal factor infertility refers to infertility caused by no factors other than adhesion, blockage, and obstruction of the fallopian tubes.

### Ovarian stimulation protocol

All included patients underwent COH using GnRH-ant flexible protocol as previously described (12). In brief, daily Gn [Recombinant Human Follitropin Alfa for Injection; Merck Serono S.p.A., Geneva, Switzerland; specification, 5.5 μg (75 IU)] was injected from the second or third day of the menstrual cycle for ovarian stimulation, with an initial dose of 125–375 IU/day, individualized according to patients’ baseline characteristics. After 4–5 days of Gn administration, the Gn dose was adjusted according to the individual patient’s response to ovarian stimulation. Follicular growth and hormone levels were monitored using transvaginal ultrasound and blood assay, respectively. Daily subcutaneous injection of GnRH-ant (Cetrorelix acetate powder for injection; Merck Serono S.p.A., Geneva, Switzerland; specification, 0.25 mg) was initiated with a dose of 0.125–0.5 mg when lead follicle size (LFS) ≥14 mm or LH ≥10 mIU/ml.

Human chorionic Gn (HCG) (Chorionic Gonadotrophin for Injection; Livzon Pharmaceutical Group Inc., Zhuhai, China; specification, 2,000 IU) of 6,000–12,000 IU was injected for final oocyte maturation when the lead follicular diameter reached 18 mm or at least three follicles had reached a diameter of 17 mm. The dose of HCG varied between 6,000 and 12,000 IU, depending on individual patient’s body mass index (BMI) and the serum E_2_ level on the trigger day. Vaginal oocyte retrieval was performed under ultrasound guidance 36–38h after HCG injection.

### Hormone analysis

Serum sex hormone levels were regularly monitored throughout the process of COH. Hormone analysis was performed at least four times: (i) day 2~3 of the menstrual cycle, (ii) day 4~5 of COH, (iii) day 6~7 of COH, and (iv) the trigger day. Fasting blood sampling from the median cubital vein was performed from 8:00 to 10:00 am. After centrifugation, serum was separated and stored at −20°C for subsequent assay. Blood FSH, LH, E_2_, and P assay was performed using the corresponding kits and the automatic microparticle chemiluminescence system (Beckman Coulter, USA). Through the immune antigen-antibody reaction, the antibody luminescence was quantified with microparticles, followed by the measurement of its photon value. Main technical indicators: intra-assay coefficient of variation <5.4%, and inter-assay coefficient of variation <10%.

### Embryo transfer and luteal phase support

Both conventional IVF and ICSI were performed for fertilization, and the choice of fertilization method depends on sperm quality. Fertilization was confirmed by observing a second polar body and two pronuclei. The cleavage stage embryos were observed 48 h after oocyte retrieval. The embryos of grades I, II, and III were selected as transplantable embryos 72 h post-insemination. Grade I embryos were defined as those with regular blastomere morphology, uniform size, no granular cytoplasm, intact zona pellucida, and a fragmentation rate of 0%–5%. Grade II embryos were those with slightly irregular blastomere morphology, slightly uneven size, granular cytoplasm, and a fragmentation rate of 6%–20%. Grade III embryos were those with irregular blastomere morphology, obviously uneven size, obviously granular cytoplasm, and a fragmentation rate of 21%–50%.

Fresh embryo transfers were performed 3 days after oocyte retrieval (D3). Cycles were converted to freeze-all strategy in patients who are at a high risk of OHSS, with peak serum progesterone >2 ng/ml during COH or with an endometrial thickness <7 mm. In the procedure of embryo transfer, the “Management Measures of Human Assisted Reproductive Technology” issued by the Ministry of Health of China was strictly abided by: during the first time of embryo transfer, women under 35 years cannot be transferred with more than two embryos, but they are allowed to be transferred with three embryos during the second time. Vaginal progesterone gel of 90 mg/day (Progesterone vaginal gel; Merck Serono S.p.A., Geneva, Switzerland; specification, 1.125 g) and oral dydrogesterone of 10 mg twice a day (Dydrogesterone; Abbott Biological B.V., Weesp, The Netherlands; specification, 10 mg) were administered for luteal phase support.

### Outcome measures

The following demographic, cycle characteristics, and pregnancy outcome data were analyzed: female age, infertility duration, type of infertility, gravidity, parity, miscarriage, cycle number, BMI, basal serum sex hormone levels, total Gn dose, duration of stimulation, endometrial thickness and sex hormonal levels on the trigger day, the number of retrieved oocytes and transferrable embryos, fertilization method, and CPR. The primary outcome was defined as clinical pregnancy, which was confirmed by the observation of the gestational sac *via* transvaginal ultrasound 30–40 days following embryo transfer. The CPR was calculated as (number of cycles with clinical pregnancy/number of all fresh transfer cycles) × 100%.

### Statistical analysis

The group differences in demographic and cycle characteristics, as well as clinical pregnancy, were examined. Continuous variables were expressed using the mean ± standard deviation (SD). Continuous variables with a normal distribution were compared using one-way ANOVA between the five groups, whereas continuous variables that were not normally distributed were compared using the Kruskal–Wallis test. For categorical variables, the count and proportion were reported, and the distribution of these variables was compared using the Pearson’s χ2 test or the Fisher’s exact test, as appropriate. Univariate logistic regression analysis was applied to analyze the potential factors affecting clinical pregnancy. Multivariate logistic regression analysis was performed to explore the independent relationship between the serum E_2_ level on the day of GnRH initiation and clinical pregnancy. A two-piecewise linear regression model was further applied to examine the threshold effect of the serum E_2_ level on the day of GnRH initiation on clinical pregnancy using a smoothing function. The threshold level (i.e., turning point) was determined using trial and error, including the selection of turning points along a pre-defined interval and then choosing the turning point that gave the maximum model likelihood. Moreover, a log-likelihood ratio test comparing the one-line linear regression model with a two-piecewise linear model was performed. All statistical analyses were performed with the statistical packages R (The R Foundation; version 3.6.1), Empower(R) (X&Y solutions, Boston, MA, USA) and IBM SPSS Statistics for Windows, version 25.0 (IBMCorp., Armonk, N.Y., USA). *P* < 0.05 was considered statistically significant.

## Results

### Baseline data, cycle characteristics, and clinical pregnancy outcomes

A total of 1,493 included cycles were divided into five distinct groups according to the quintile serum E_2_ levels on the day of GnRH-ant initiation: Q1: <436.8 pg/ml (n = 298), Q2: 436.8–658.6 pg/ml (n = 299), Q3: 658.6–894.4 pg/ml (n = 299), Q4: 894.4–1359.0 pg/ml (n = 298), and Q5: >1,359.0 pg/ml (n = 299) (**Supplemental Figure 1**). The comparison of baseline data, cycle characteristics, and clinical pregnancy outcomes among the five groups was presented in **Table 1**. As for the baseline data, the five groups were comparable in regard to age, infertility duration, cycle number, BMI, and basal E_2_ levels. However, antral follicle count (AFC) increased significantly from the lowest quintile (Q1) to the highest quintile (Q5) (*P* < 0.001).

**Table 1:** Baseline data, cycle characteristics, and clinical pregnancy outcomes of the patients based on the serum E_2_ levels on the day of GnRH-ant administration.

Serum E_2_ on the day of GnRH-ant initiation	Q1 (E_2_ < 436.8)	Q2 (436.8 ≤ E_2_ < 658.6)	Q3 (658.6 ≤ E_2_ < 894.4)	Q4 (894.4 ≤ E_2_ < 1359.0)	Q5 (E_2_ ≥ 1359.0)	P-value
N	298	299	299	298	299	
Age, years	33.24 ± 4.32	32.99 ± 5.15	33.13 ± 4.79	32.63 ± 4.90	32.34 ± 3.77	0.097
Infertility duration, years	4.49 ± 2.81	4.40 ± 3.51	4.26 ± 3.31	4.13 ± 3.54	4.07 ± 3.20	0.056
Cycle number	1.81 ± 0.90	1.79 ± 1.24	1.71 ± 1.02	1.67 ± 0.84	1.64 ± 0.79	0.211
BMI, kg/m^2^	24.05 ± 3.33	23.84 ± 3.58	24.08 ± 9.24	23.43 ± 3.22	23.04 ± 3.60	0.077
Basal E_2_, pg/ml	48.89 ± 45.25	54.26 ± 69.21	47.93 ± 41.27	45.14 ± 32.90	46.82 ± 39.37	0.841
AFC	7.87 ± 4.89	8.84 ± 4.96	9.35 ± 5.10	11.19 ± 6.18	12.07 ± 6.28	<0.001
Total Gn dose, IU	2,589.10 ± 1,012.79	2,562.17 ± 772.82	2,556.66 ± 832.98	2,462.72 ± 703.26	2,283.48 ± 738.81	<0.001
Duration of stimulation, days	9.24 ± 2.55	9.31 ± 1.83	9.37 ± 1.95	9.50 ± 1.66	9.31 ± 1.69	0.585
Cycle day on the day of GnRH-ant initiation	6.27 ± 2.49	6.80 ± 1.46	7.13 ± 1.66	7.24 ± 1.58	7.69 ± 1.80	<0.001
LFS on the day of GnRH-ant initiation	13.53 ± 3.43	14.66 ± 2.23	14.89 ± 2.35	15.16 ± 2.52	16.23 ± 2.56	<0.001
Serum E_2_ on trigger day, pg/ml	1,258.64 ± 1,133.32	1,776.03 ± 1,110.81	2,234.94 ± 1,214.90	2,877.17 ± 1,439.55	3,555.61 ± 1,350.88	<0.001
Serum P on trigger day, ng/ml	0.91 ± 0.49	0.99 ± 0.56	1.06 ± 0.47	1.16 ± 0.52	1.21 ± 0.50	<0.001
Endometrial thickness on trigger day, mm	9.97 ± 1.89	10.44 ± 1.98	10.29 ± 1.84	10.23 ± 1.68	10.46 ± 1.81	0.009
Fertilization method						0.347
IVF	282 (94.63%)	272 (90.97%)	278 (92.98%)	284 (95.30%)	283 (94.65%)	
ICSI	15 (5.03%)	25 (8.36%)	21 (7.02%)	14 (4.70%)	15 (5.02%)	
IVF/ICSI	1 (0.34%)	2 (0.67%)	0 (0.00%)	0 (0.00%)	1 (0.33%)	
Oocytes retrieved	4.81 ± 3.59	6.45 ± 3.54	7.16 ± 3.74	9.29 ± 4.48	11.20 ± 4.91	<0.001
Transferable embryos	2.02 ± 1.20	2.53 ± 1.33	2.65 ± 1.50	2.90 ± 1.59	3.18 ± 1.85	<0.001
Transferred embryos	1.67 ± 0.56	1.87 ± 0.46	1.85 ± 0.46	1.93 ± 0.47	1.87 ± 0.47	<0.001
Clinical pregnancy, % (n)	112 (37.58%)	144 (48.16%)	130 (43.48%)	114 (38.26%)	124 (41.47%)	0.049

Q1–5, quintile 1–5; E2, estradiol; BMI, body mass index; AFC, antral follicle count; Gn, gonadotropin; LFS, lead follicle size; P, progesterone; IVF, *in vitro* fertilization; ICSI, intracytoplasmic sperm injection.

As for the cycle characteristics, the duration of ovarian stimulation and fertilization method were comparable among the five groups. From the lowest quintile (Q1) to the highest quintile (Q5), cycle day and LFS on the day of GnRH-ant administration, serum E_2_, and progesterone (P) on trigger day as well as the number of retrieved oocytes and transferrable embryos increased significantly (*P* < 0.001), whereas the total dose of Gn used during COH decreased significantly (*P* < 0.001). Endometrial thickness and the number of transferred embryos were also significantly different among the five groups (*P =* 0.009 and < 0.001, respectively).

Statistically significant difference was found in clinical pregnancy among the five groups (*P =* 0.06), with a maximum of 48.16% in the second quintile (Q2).

### Serum E2 on the day of GnRH-ant initiation was an independent predictor of clinical pregnancy by univariate and multivariate logistic regression analysis

Univariate logistic regression analysis demonstrated that serum E_2_ on the day of GnRH-ant initiation had a statistically significant effect on clinical pregnancy (**Table 2**). In addition, age and cycle number had a statistically significant negative effect on clinical pregnancy, whereas AFC, endometrial thickness on trigger day, and the number of transferred embryos had a statistically significant positive effect on clinical pregnancy.

**Table 2:** Univariate logistics regression analysis for clinical pregnancy.

	OR (95% CI)	P-value
Age	0.93 (0.92, 0.95)	<0.0001
Infertility duration	0.98 (0.95, 1.00)	0.0882
Cycle number	0.83 (0.76, 0.92)	0.0002
BMI	0.99 (0.98, 1.01)	0.4706
Basal E_2_	1.00 (1.00, 1.00)	0.2179
AFC	1.04 (1.02, 1.06)	<0.0001
Total Gn dose	1.00 (1.00, 1.00)	0.1825
Duration of stimulation	1.01 (0.96, 1.05)	0.8144
Endometrial thickness on trigger day	1.08 (1.03, 1.13)	0.0020
Oocytes retrieved	1.02 (1.00, 1.04)	0.1267
Transferred embryos	1.43 (1.19, 1.73)	0.0001
Cycle day on the day of GnRH-ant initiation	1.00 (0.95, 1.06)	0.9110
LFS on the day of GnRH-ant initiation	1.02(0.98, 1.06)	0.3554
Serum E_2_ on trigger day, pg/ml	1.00(1.00, 1.00)	0.2488
Serum P on trigger day, ng/ml	0.94(0.77, 1.15)	0.5721
Fertilization method		
IVF	1.0	
ICSI	1.24 (0.86, 1.79)	0.2496
IVF/ICSI	1.42 (0.28, 7.03)	0.6712
Serum E_2_ on the day of GnRH-ant initiation		
Q1 (E2 < 436.8)	0.65 (0.47, 0.90)	0.0092
Q2 (436.8 ≤ E2 < 658.6)	1	
Q3 (658.6 ≤ E2 < 894.4)	0.83 (0.60, 1.14)	0.2507
Q4 (894.4 ≤ E2 < 1359.0)	0.67 (0.48, 0.92)	0.0148
Q5 (E2 ≥ 1359.0)	0.76 (0.55, 1.05)	0.1003

BMI, body mass index; AFC, antral follicle count; E2, estradiol; Gn, gonadotropin; LFS, lead follicle size; P, progesterone; IVF, *in vitro* fertilization; ICSI, intracytoplasmic sperm injection; Q1–5, quintile 1–5.

The effect of serum E_2_ on the day of GnRH-ant initiation was further analyzed using multivariate logistic regression after adjusting for the confounding factors including age, cycle number, infertility duration, BMI, AFC, and number of transferred embryos. The result showed that serum E_2_ on the day of GnRH-ant initiation was an independent predictor for clinical pregnancy (**Table 3**). Compared with the second quintile (Q2), the odds ratio (OR) of CPR in the first (Q1) and third (Q3) quintiles were 0.77 (95% CI: 0.53, 1.10) and 0.73 (95% CI: 0.51, 1.04), respectively, but the difference was not statistically significant (*P =* 0.1523 and 0.0819, respectively). Furthermore, compared with the second quintile (Q2), the clinical pregnancy in the fourth (Q4) and fifth quintiles (Q5) decreased significantly [OR = 0.57 (95% CI: 0.39, 0.82), *P* = 0.0025 and OR = 0.56 (95% CI: 0.39, 0.80), *P* = 0.0015, respectively].

**Table 3:** Adjusted effect of the serum E2 level on the day of GnRH-ant initiation on clinical pregnancy.

	*Adjusted, ß/OR (95% CI)	P-value
Clinical pregnancy		
Serum E_2_ on the day of GnRH-ant initiation		
Q1 (E_2_ < 436.8)	0.77 (0.53, 1.10)	0.1523
Q2 (436.8 ≤ E_2_ < 658.6)	1	
Q3 (658.6 ≤ E_2_ < 894.4)	0.73 (0.51, 1.04)	0.0819
Q4 (894.4 ≤ E_2_ < 1359.0)	0.57 (0.39, 0.82)	0.0025
Q5 (E_2_ ≥ 1359.0)	0.56 (0.39, 0.80)	0.0015

OR, odds ratio; E2, estradiol. *Adjusted for age, cycle number, infertility duration, body mass index, antral follicle count, and number of transferred embryos.

### The optimal level of serum E2 on the day of GnRH-ant initiation was determined as 498 pg/ml by smooth curve fitting and threshold analysis

**Figure 2:**
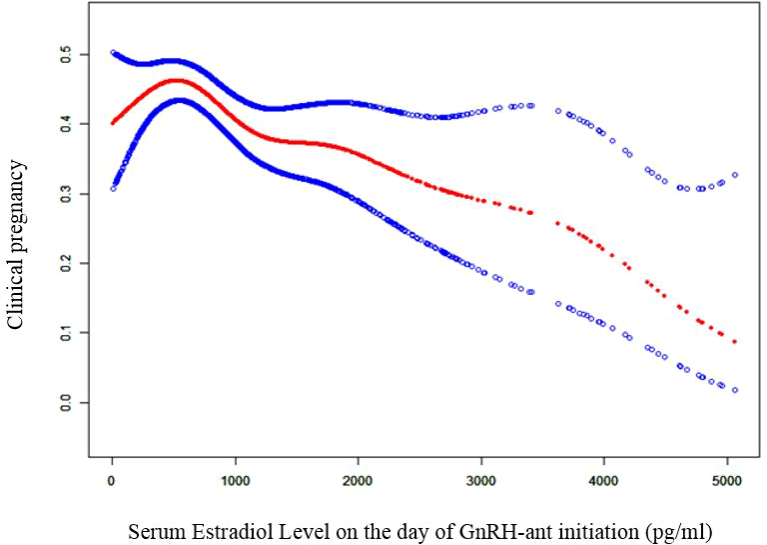
Curve fitting diagram of serum estradiol level on GnRH-ant start day and clinical pregnancy. Age, infertility duration, body mass index, cycle number, AFC, and the number of transferred embryos were used as adjusted variables when analyzing the relationship between serum estradiol level on the day of GnRH-ant initiation and clinical pregnancy. GnRH-ant, gonadotropin releasing hormone antagonist.

To evaluate the non-liner relationship between serum E2 on the day of GnRH-ant initiation and the CPR, a fitting curve was applied (**Figure 2**). A two-piecewise linear regression model was further applied to examine the threshold effect of serum E_2_ on the day of GnRH-ant initiation on CPR. The result of smooth curve fitting revealed that a non-linear relationship existed between serum E_2_ on the day of GnRH-ant initiation and the CPR after adjusting for age, infertility duration, BMI, cycle number, AFC, and the number of transferred embryos. With the increase of serum E_2_ on the day of GnRH-ant initiation, the CPR showed a trend of slight increase and then slight decrease (**Figure 2**). We further performed threshold analysis and adjusted for the same confounding confounding factors as what were adjusted in smooth curve fitting, and the turning point was determined as 498 pg/ml (**Table 4**). When serum E_2_ was lower than 498 pg/ml, the OR of clinical pregnancy was 1.05 (95% CI: 1.00, 1.11, *P =* 0.0583). When serum E_2_ was higher than 498 pg/ml, the OR of clinical pregnancy was 0.97 (95% CI: 0.95, 0.98, *P =* 0.0003) (**Table 4**).

**Table 4:** Threshold effect analysis of serum estradiol level on the day of GnRH-ant initiation on clinical pregnancy using piecewise linear regression.

	Crude ß/OR (95% CI)	P-value	*Adjusted ß/OR (95% CI)	P-value
**Clinical pregnancy**				
E_2_ < 4.98 (100 pg/ml)	0.99 (0.92, 1.07)	0.7979	1.05 (1.00, 1.11)	0.0583
E_2_ ≥ 4.98 (100 pg/ml)	0.98 (0.96, 1.00)	0.0333	0.97 (0.95, 0.98)	0.0003

Crude: no adjustment. *Adjusted for age, infertility duration, body mass index, cycle number, antral follicle count, and number of transferred embryos.

## Discussion

This retrospective cohort study showed that, among patients diagnosed with simple tubal factor infertility using the GnRH-ant protocol for COH followed by fresh embryo transfer, the serum E_2_ level on the day of GnRH-ant initiation was an independent predictor of clinical pregnancy. This indicated that the E_2_ level could serve as an adjuvant parameter to determine when to initiate GnRH-ant administration. We further revealed a threshold effect based on CPR. To the best of our knowledge, this is the first retrospective cohort study to reveal a clear nonlinear association between the serum E_2_ level on the day of GnRH-ant initiation and CPR of fresh embryo transfer cycles.

Our study demonstrated that the best E_2_ value with the maximal CPR after fresh embryo transfer was 498 pg/ml. If GnRH-ant was initiated when E_2_ was below 498 pg/ml, then the CPR increased by 5% with every 100 pg/ml increase in E_2_ level, but this increase was marginally significant. If GnRH-ant was initiated when E_2_ was above 498 pg/ml, then the CPR decreased by 3% with every 100 pg/ml increase in E_2_ level, which was with statistical significance. Given that the increasing and decreasing trends of CPR with E_2_ were mild, the CPR remained high if GnRH-ant was initiated when E_2_ was between 436.8 and 658.6 pg/ml. However, when E_2_ was above 894.4 pg/ml, the CPR reduced significantly by more than 40%. Our finding is, in part, consistent with a previous study by Schumacher et al., which demonstrated that the highest CPR was achieved when GnRH-ant was initiated when the E_2_ level was 500-599 pg/ml and initiating GnRH-ant when the E_2_ level was above 1,100 pg/ml was associated with a reduction by 40% in CPR (13). However, our study does not support its finding that initiating GnRH-ant when E_2_ level was below 300 pg/ml also led to a significant reduction in CPR (13). In their study, many confounding factors including infertility duration, endometrial thickness on trigger day, and the number of transferred embryos were not adjusted. This may bias their findings.

We speculated that the significant reduction in CPR of cycles, of which GnRH-ant was initiated when serum E_2_ was above 894.4 pg/ml, was due to the impacted endometrial receptivity caused by supraphysiologic hormone milieu during COH. Our data showed that the serum E_2_ and P levels on the trigger day were higher in groups with higher serum E_2_ levels on the day of GnRH-ant initiation, suggesting that the cycles with high serum E_2_ levels on the day of GnRH-ant initiation were exposed to high E_2_ and P levels throughout the COH process. Some previous studies have demonstrated that supraphysiologic E_2_ generated by COH was associated with a detrimental effect on endometrial receptivity and CPR (10, 14–16). Other studies demonstrating the possible association between high E_2_ and preeclampsia, low birth weight, and small for gestational age (8, 9) also support the hypothesis that high E_2_ level is associated with abnormal endometrial decidualization and suboptimal placentation. One possible mechanism is that supraphysiological E_2_ may result in endometrial edema, which hinders uteroplacental blood flow modulation and villous trophoblast invasion (17). Similarly, there is accumulating evidence that increased P levels at the end of the follicular phase may lead to a shift in the implantation window (18) and are detrimental to clinical outcomes (19–22).

Supraphysiological E_2_ levels could also be associated with an adverse effect on oocyte/embryo quality. Although we found that the number of oocytes retrieved and transferrable embryos increased with the increase of serum E_2_ levels on the day of GnRH-ant initiation, the growth in the number of transferrable embryos appeared to be smaller than that in retrieved oocytes. Assuming that only mature oocytes have the potential of being fertilized and becoming transferrable embryos, the difference in the growth of the numbers of transferrable embryos and retrieved oocytes could probably be explained by suboptimal follicular synchronization and impaired oocyte maturation rate associated with high E_2_ levels on the day of GnRH-ant initiation (23). Moreover, *in vitro* experiments revealed that cell proliferation in human embryonic stem cells was suppressed when they were exposed to supraphysiologic levels of E_2_ (24).

According to the “two-cell, two-gonadotropin” model (25), suppression of endogenous LH induced by GnRH-ant leads to the reduced biosynthetic activity of the theca cells, and thus, there is a lower amount of androgen substrate for conversion into E_2_ by aromatase and 17b-hydroxysteroid dehydrogenase in granulosa cells under the modulation of FSH. Thus, it is reasonable to speculate that initiating GnRH-ant when serum E_2_ is low is likely to restrict E_2_ biosynthesis during the mid-late phase of COH. One randomized controlled trial supported this speculation by reporting a more physiologic E_2_ level during COH when GnRH-ant was initiated on cycle day 2 compared with day 6 (26). Thus, it is reasonable that adding GnRH-ant according to an E_2_ threshold may ameliorate the hormonal profile during COH and mitigate its consequent adverse effects on both endometrium receptivity and oocyte/embryo quality.

The strength of our study was that we analyzed a large cohort of 1,493 cycles using a sophisticated mathematic model to reveal a clear non-linear relationship and determine the turning point. Although our study was retrospective in nature, we adjusted for a series of confounding factors involving baseline characteristics and cycle-specific ovarian stimulation and embryo transfer parameters so that the bias has been minimized. However, many patients with high E_2_ levels yielding a satisfying number of transferrable and good quality embryos adopted a freeze-all strategy. As we only included fresh transfer cycles, most patients with high E_2_ levels receiving fresh embryo transfer were those with a suboptimal number and quality of embryos. This could bias the CPR of high estradiol groups. As a result, future prospective studies and randomized controlled trials are warranted to investigate whether serum E_2_ alone could be used as a parameter for GnRH-ant initiation and to determine its optimal criteria. In addition, prospective trials comparing CPR after fresh and frozen embryo transfer among patients with high E_2_ levels on the day of GnRH-ant initiation may reveal whether the high estradiol-associated CPR reduction is mediated by impaired endometrial receptivity or damaged oocyte/embryo quality.

## Conclusions

In conclusion, our study demonstrates that the serum E_2_ level should be considered as a parameter for GnRH-ant initiation in addition to lead follicle diameter and serum LH level. Although the best E_2_ value for GnRH-ant initiation is determined as 498 pg/ml, GnRH-ant could be recommended to initiate when serum E_2_ is in an optimal range (436.8–658.6 pg/ml). Notably, if GnRH-ant is initiated when serum E_2_ is above 894.4 pg/ml, then the CPR following fresh embryo transfer could drop dramatically. Thus, cancellation of fresh embryo transfer and starting GnRH-ant earlier in the future cycles should be suggested.

## Data availability statement

The original contributions presented in the study are included in the article/**Supplementary Material**. Further inquiries can be directed to the corresponding author.

## Ethics statement

The studies involving human participants were reviewed and approved by the ethics committee of the Second Hospital of Hebei Medical University. Written informed consent for participation was not required for this study in accordance with the national legislation and the institutional requirements.

## Author contributions

The study was designed by YW and XX. The relevant clinical data were extracted by JZ and A-mY. YW, Z-yL, YH, NC, and Z-mZ contributed to data analysis. The manuscript was written by YW and XX and revised by Z-mZ, QL and BS. The study was conducted under the supervision of G-mH and Z-m Zhao. All authors contributed to the article and approved the submitted version.

## Funding

This study was supported by National Key R&D Program of China (2021YFC2700605). Hebei Natural Science Foundation (H2022206019, 19JCZDJC65000(Z), H2019206707, H2019206712). S&T Program of Hebei (20377714D,21377720D,21377721D), Innovation Capability Enhancement Program of Hebei Province (Hebei Clinical Medical Research Center Special Project) (20577710D). Medical Science Research Project of Hebei province (20211494).

## Acknowledgments

The authors thank all the patients who participated in this study.

## Conflict of interest

The authors declare that the research was conducted in the absence of any commercial or financial relationships that could be construed as a potential conflict of interest.

## Publisher’s note

All claims expressed in this article are solely those of the authors and do not necessarily represent those of their affiliated organizations, or those of the publisher, the editors and the reviewers. Any product that may be evaluated in this article, or claim that may be made by its manufacturer, is not guaranteed or endorsed by the publisher.

## Supplementary material

Supplementary Figure 1 Descriptive analysis of the serum estradiol level on the day of GnRH-ant initiation. GnRH-ant, gonadotropin releasing hormone antagonist.
