# Supplementary figures and images for: Optimizing estradiol level for gonadotrophin-releasing hormone antagonist initiation among patients with simple tubal factor infertility
Source: Front Endocrinol (Lausanne). 2022 Sep 9;13:915923. doi: 10.3389/fendo.2022.915923 (PMC9500406; doi:10.3389/fendo.2022.915923)

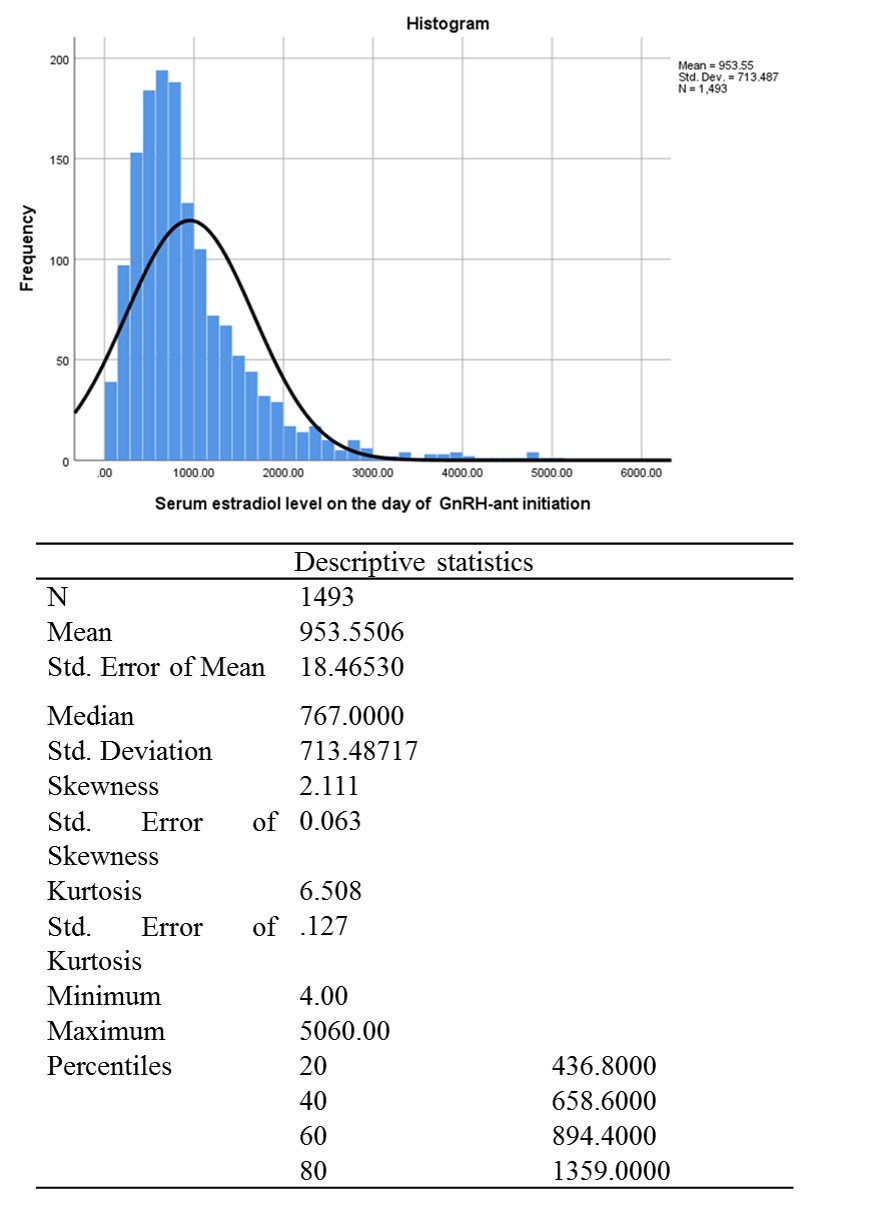

Supplement: Supplementary Figure 1 — Descriptive analysis of the serum estradiol level on the day of GnRH-ant initiation. GnRH-ant, gonadotropin releasing hormone antagonist. [file Image_1.jpg]
